# The LIN28/let-7 Pathway in Cancer

**DOI:** 10.3389/fgene.2017.00031

**Published:** 2017-03-28

**Authors:** Julien Balzeau, Miriam R. Menezes, Siyu Cao, John P. Hagan

**Affiliations:** Department of Neurosurgery, University of Texas Health Science Center at HoustonHouston, TX, USA

**Keywords:** Lin28, let-7, microRNAs, cancer stem cells, proto-oncogene proteins

## Abstract

Among all tumor suppressor microRNAs, reduced let-7 expression occurs most frequently in cancer and typically correlates with poor prognosis. Activation of either LIN28A or LIN28B, two highly related RNA binding proteins (RBPs) and proto-oncogenes, is responsible for the global post-transcriptional downregulation of the let-7 microRNA family observed in many cancers. Specifically, LIN28A binds the terminal loop of precursor let-7 and recruits the Terminal Uridylyl Transferase (TUTase) ZCCHC11 that polyuridylates pre-let-7, thereby blocking microRNA biogenesis and tumor suppressor function. For LIN28B, the precise mechanism responsible for let-7 inhibition remains controversial. Functionally, the decrease in let-7 microRNAs leads to overexpression of their oncogenic targets such as MYC, RAS, HMGA2, BLIMP1, among others. Furthermore, mouse models demonstrate that ectopic LIN28 expression is sufficient to drive and/or accelerate tumorigenesis via a let-7 dependent mechanism. In this review, the LIN28/let-7 pathway is discussed, emphasizing its role in tumorigenesis, cancer stem cell biology, metabolomics, metastasis, and resistance to ionizing radiation and several chemotherapies. Also, emerging evidence will be presented suggesting that molecular targeting of this pathway may provide therapeutic benefit in cancer.

## Introduction

MicroRNAs (miRNAs) are small non-coding RNAs (~19–22 nucleotides in length) that play critical roles in post-transcriptional gene regulation (reviewed in Hagan and Croce, 2007; Lin and Gregory, 2015b; Connerty et al., 2016). In general, microRNAs are generated by a two-step process where the primary microRNA (pri-miR) transcript is cleaved in the nucleus by the Microprocessor complex to generate precursor microRNA (pre-miR) with a characteristic stem-loop structure. The pre-miR is exported to the cytoplasm where Dicer is responsible for generating the mature miRNA that is incorporated into the microRNA-induced silencing complex (miRISC). To exert their biological effect, microRNAs most frequently bind with imperfect complementarity to the 3′ UTR of their targets mRNAs leading to mRNA decay and/or translational inhibition. Studies using genetically engineered mouse models have revealed that miRNAs serve diverse functions in developmental and physiological processes.

Dysregulated microRNA expression is associated with and functionally significant for many diseases including cancer. Of note, a recent study systemically evaluated the prognostic value of miRNA expression in human cancers (Nair et al., 2012). Specifically, decreased expression of the tumor suppressor let-7 microRNAs and increased expression of oncogenic miR-21 are most frequently associated with poor prognosis as determined by analysis of 46 studies on 20 different types of human cancers. The coordinate repression of the let-7 microRNA family is striking for two reasons. First, there are 12 members of the let-7 microRNA family located on eight chromosomes, where most cells express a handful of let-7 family members. Secondly, primary let-7 transcripts are still being actively transcribed in many cancers as well as in embryonic stem cells; however, mature let-7 is not being produced as expected. Ultimately, two highly conserved RNA-binding proteins, LIN28A and LIN28B, were shown to inhibit biogenesis of mammalian let-7 miRNAs through direct binding to either pre-let-7 and/or pri-let-7 (Heo et al., 2008, 2009; Newman et al., 2008; Rybak et al., 2008; Viswanathan et al., 2008; Hagan et al., 2009; Piskounova et al., 2011). Strikingly, we showed that inhibition of either LIN28A or LIN28B via siRNA led to regression of established human xenograft tumors in mice that expressed the targeted gene (Piskounova et al., 2011). This result was recapitulated by restoring let-7 expression using a microRNA mimic, implicating let-7 dependent functions of LIN28AB in tumor regression. For clarity throughout this review, LIN28AB will be used to refer collectively to both LIN28A and LIN28B. Also, it is worth noting that many publications refer to LIN28 (the official gene name for LIN28A) when in reality the study involved LIN28B.

In addition to miRNAs, cancer stem cells (CSCs) represent another important target for the design of novel anticancer treatments. Unlike differentiated cancer cells present in many tumors, CSCs are a rare tumor cell population that has stem cell-like properties and are sufficient in small numbers to regrow tumors. They have the ability of self-renewal and can differentiate into heterogeneous types of tumor cells (O'Connor et al., 2014). While frontline cancer treatments are often effective against the bulk of the tumor, most chemotherapeutic drugs show poor efficacy against CSCs. Considerable evidence has shown that CSCs is one of the underlying mechanisms of tumor resistance to chemotherapy and radiation therapy and are often responsible for recurrence of more deadly disease. Not surprisingly, expression of LIN28A or LIN28B serves as CSC biomarkers in multiple cancers (Zhou et al., 2013). LIN28AB directly contributes to the regulation and maintenance of stemness of CSCs. Moreover, LIN28AB via a let-7 dependent mechanism confers resistance to ionizing radiation and several chemotherapies.

## Structure and key biological functions of LIN28A and LIN28B

**Figure 1:**
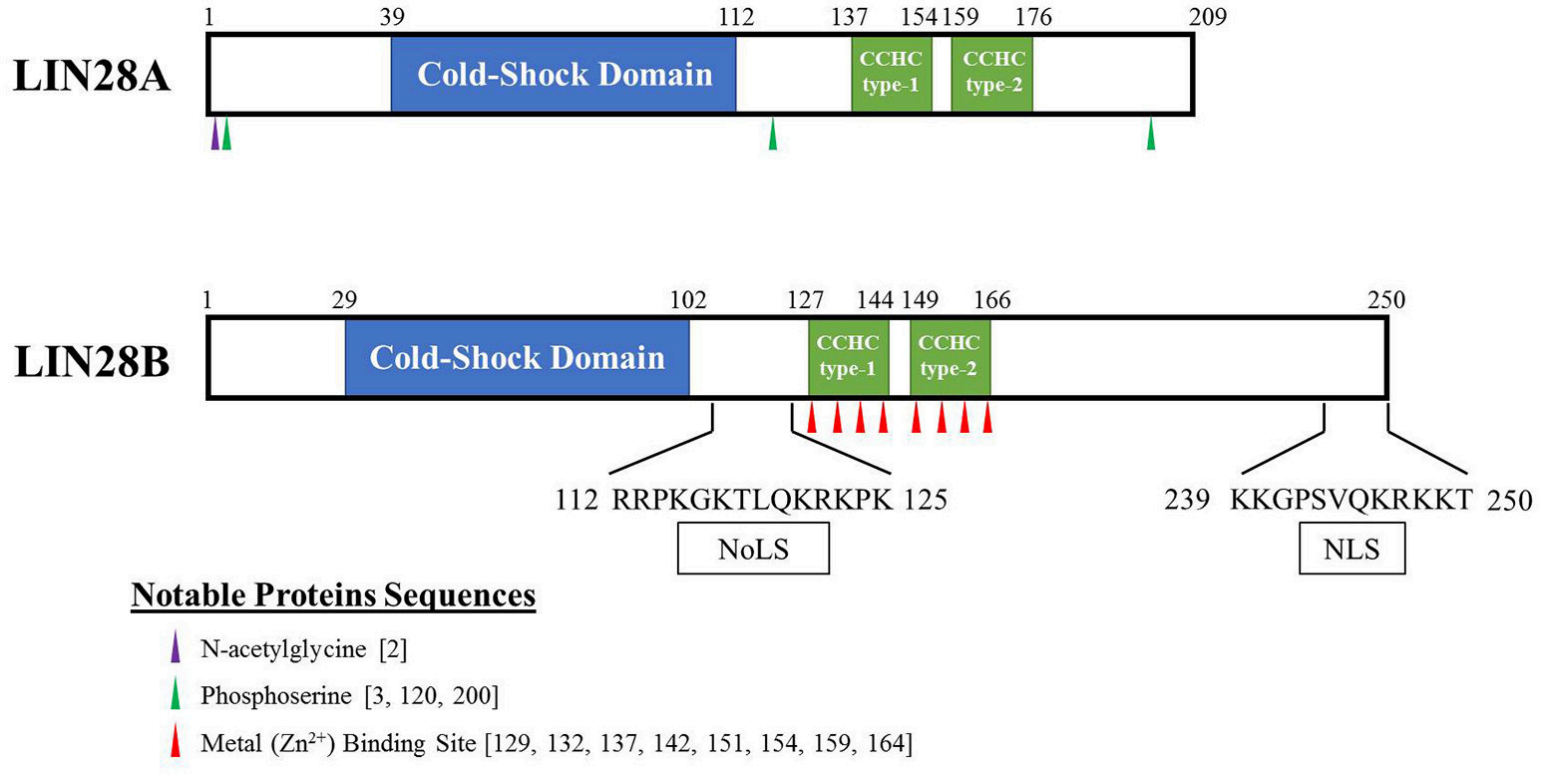
**Schematic of human LIN28A and LIN28B proteins**. These highly related proto-oncogenes have two distinct RNA binding regions. The first is a cold-shock domain (highlighted in blue) with preference for GNGAY RNA sequences while the CCHC Zn fingers (highlighted in green) bind preferentially to a GRAG motif (R = G or A). Binding to both RNA sites is required for high affinity pre-let-7 binding. Putative nucleolar localization signal (NoLS) and nuclear localization signal (NLS) are reported for LIN28B.

**Figure 2:**
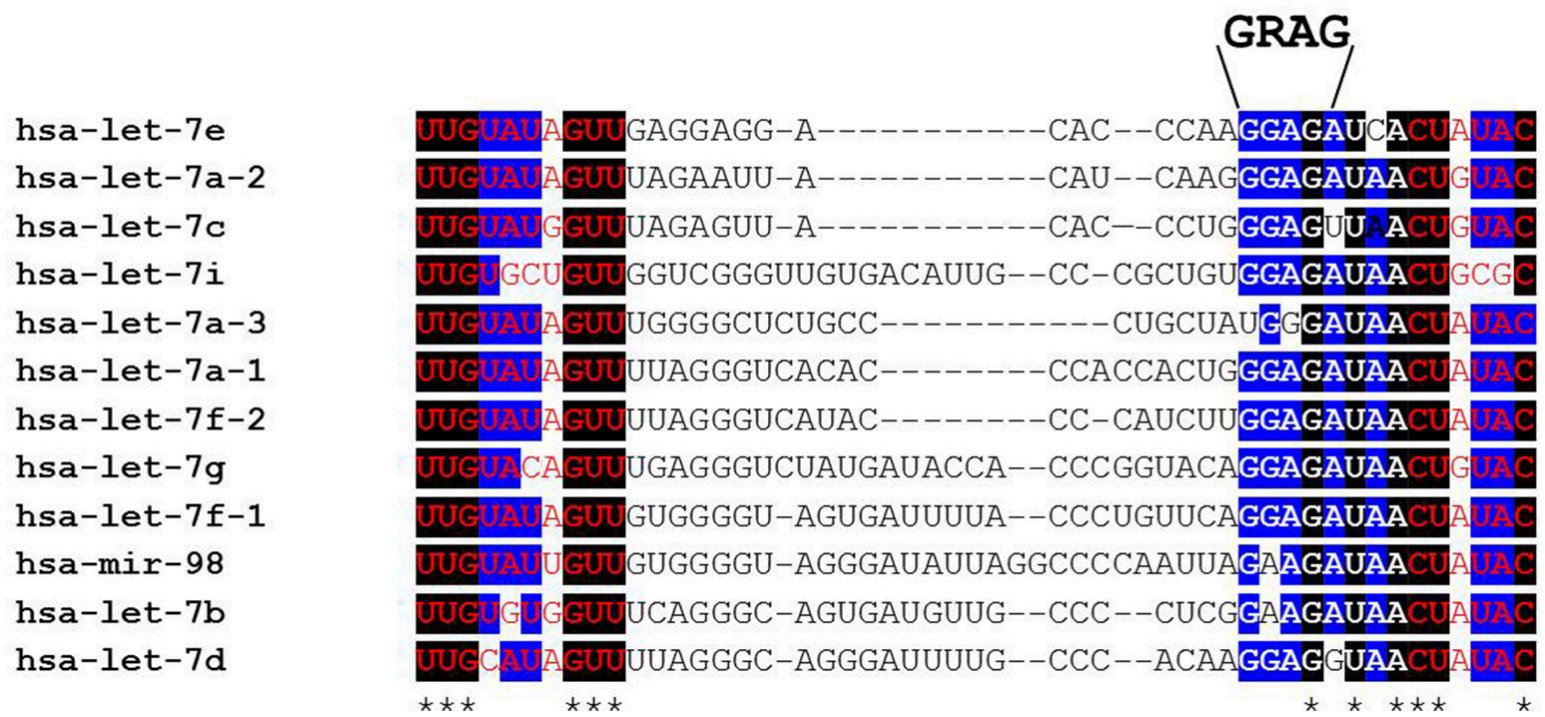
**Alignment of human pre-let-7 sequences (partial) with a focus on the terminal loop**. The bases shown in red font are part of the let-7-5p/let-7-3p microRNA duplex following cytoplasmic Dicer cleavage. Black boxes and asterisks denote perfectly conserved bases while blue boxes represent bases where 10/12 are identical across all let-7 family members. Note that the GRAG (9 GGAG > 2 GAAG) motif is conserved across all family members except human let-7a-3 that is reported to escape LIN28AB-mediated repression. This RNA sequence motif is bound by the CCHC zinc fingers while the cold shock domain binds the GNGAY motif (and close variants) that lies at varying distances 5′ of the GGAG motif.

Lin-28 and let-7 were first discovered and studied in the nematode *Caenorhabditis elegans* as heterochronic genes that regulate developmental timing (Moss et al., 1997; Reinhart et al., 2000; Slack et al., 2000). In worms to mammals, Lin28 blocks let-7 expression, while let-7 itself binds to the 3′ UTR of Lin28 mRNA to regulate negatively Lin28 expression, thereby establishing a double negative feedback loop. In mammals, there are two LIN28 family members. The human LIN28A and LIN28B genes encode 209- and 250-amino acid proteins, respectively (Figure 1). Both RNA binding proteins (RBPs) share a high degree of homology in structural domains with an N-terminal cold-shock domain (CSD) and two C-terminal CysCysHisCys (CCHC) zinc finger domains (Guo et al., 2006). Crystallography and biochemical studies demonstrate that the CSD and Zinc finger domains bind GNGAY and GGAG motifs in the pre-let-7 terminal loop, respectively (Nam et al., 2011; Figure 2). The CSD and Zinc finger domains are separated by a flexible region that permits binding to distinct pre-let-7 loops where the GNGAY and GGAG motifs vary in their spacing. Protein interactions with both RNA sequence motifs are required for high affinity binding.

**Figure 3:**
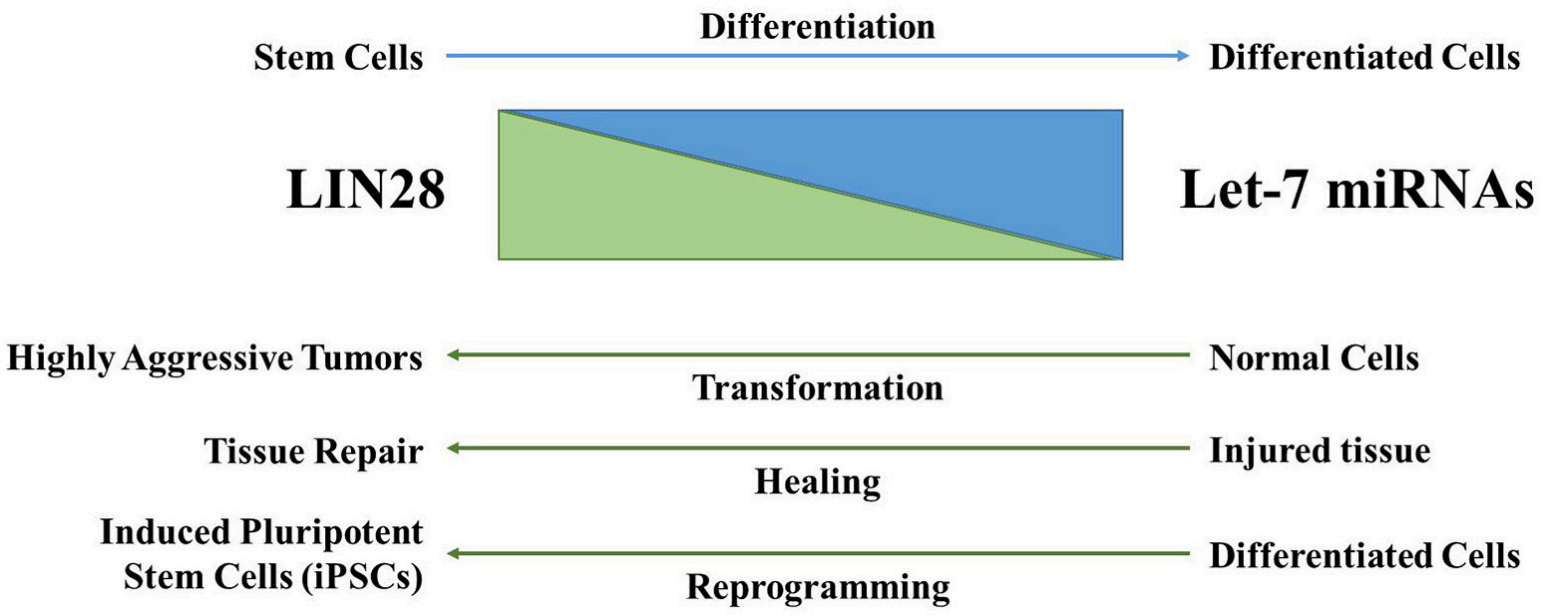
**The LIN28AB/let-7 axis has significant biological functions**. LIN28AB expression is high in undifferentiated cells where these proteins block biogenesis of the let-7 microRNA family. As differentiation progresses, LIN28AB expression is lost, resulting in production of mature let-7 microRNAs that themselves negatively regulate LIN28AB. During cellular transformation, wound healing (in particular in young mammals), and in the generation of iPSCs, LIN28AB helps drive pluripotency, self-renewal, de-differentiation, and/or cellular transformation.

To unravel the *in vivo* function of Lin28ab in mammals, we in collaboration with the Daley lab generated and characterized conditional mouse knockouts of both genes (Shinoda et al., 2013). Lin28a null mice experience early perinatal lethality, while Lin28b knockout leads to postnatal growth defects solely in males. Double knockout causes embryonic lethality by E13 suggesting that these proteins have functional redundancy during development. Overall, Lin28a and Lin28b expression is largely restricted to embryonic development in mammals. Consistent with this observation, conditional mouse knockout of either gene at 6 weeks of age in mice yields no overt phenotypes. During differentiation, the levels of Lin28AB are markedly reduced with a concomitant increase in let-7 microRNAs (Mayr and Heinemann, 2013; Figure 3). LIN28A is predominantly/almost exclusively cytoplasmic and has been detected in association with ribosomes, P-bodies, and stress granules (Balzer and Moss, 2007). For LIN28B, there are divergent opinions with regards to its subcellular localization. A report found that LIN28B has distinct nucleolar and nuclear localization signals (Piskounova et al., 2011), while other research found that LIN28B is predominantly cytoplasmic and suggest that it may shuttle into the nucleus in a cell-cycle dependent manner (Guo et al., 2006; Molenaar et al., 2012; Hafner et al., 2013). Further studies are required to elucidate fully the exact subcellular location of human LIN28B that may vary dependent on cell type. This knowledge will inform the precise mechanism(s) by which LIN28B blocks let-7 biogenesis.

**Figure 4:**
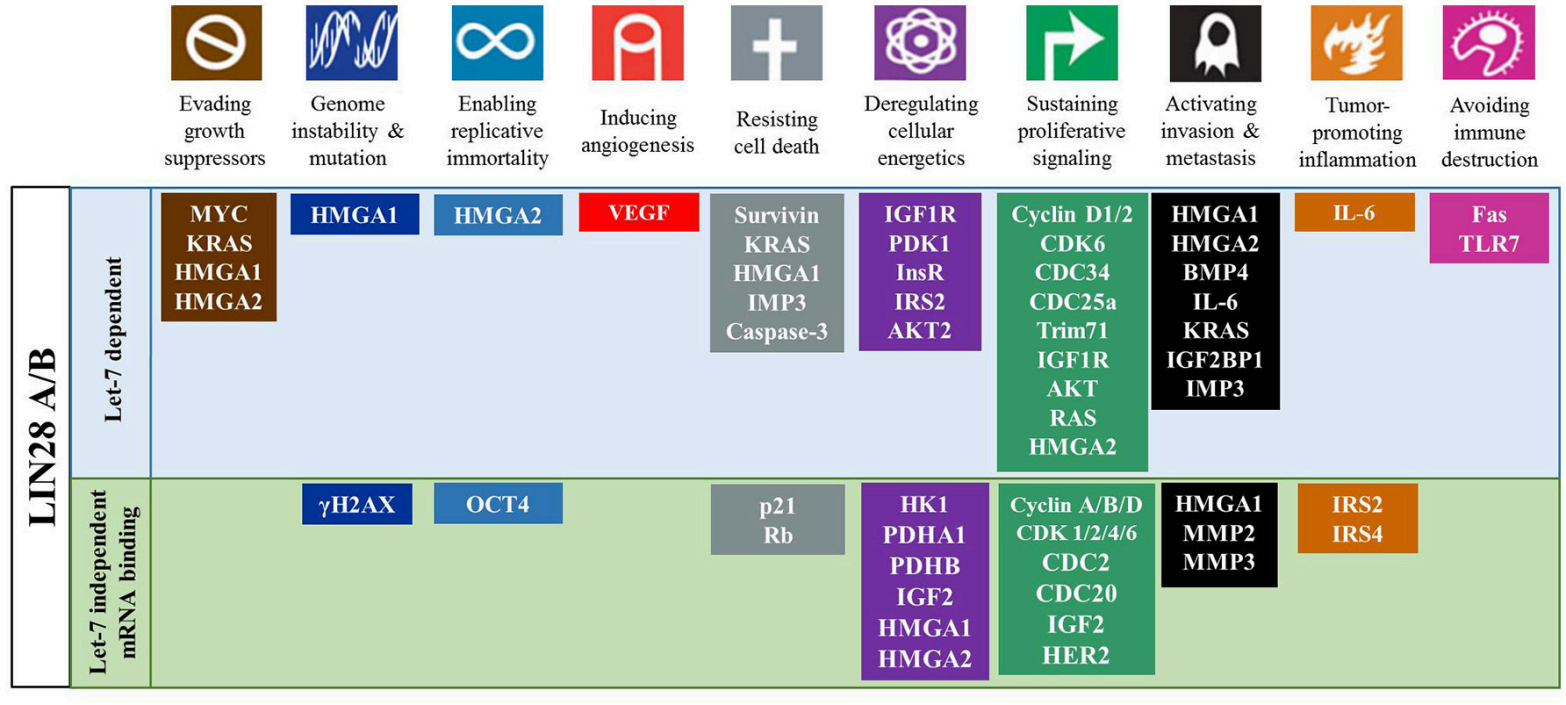
**The LIN28AB/let-7 axis has significant impact on cancer hallmarks**. LIN28AB through let-7 dependent and independent mechanisms promote multiple processes that promote cancer development and progression.

**Figure 5:**
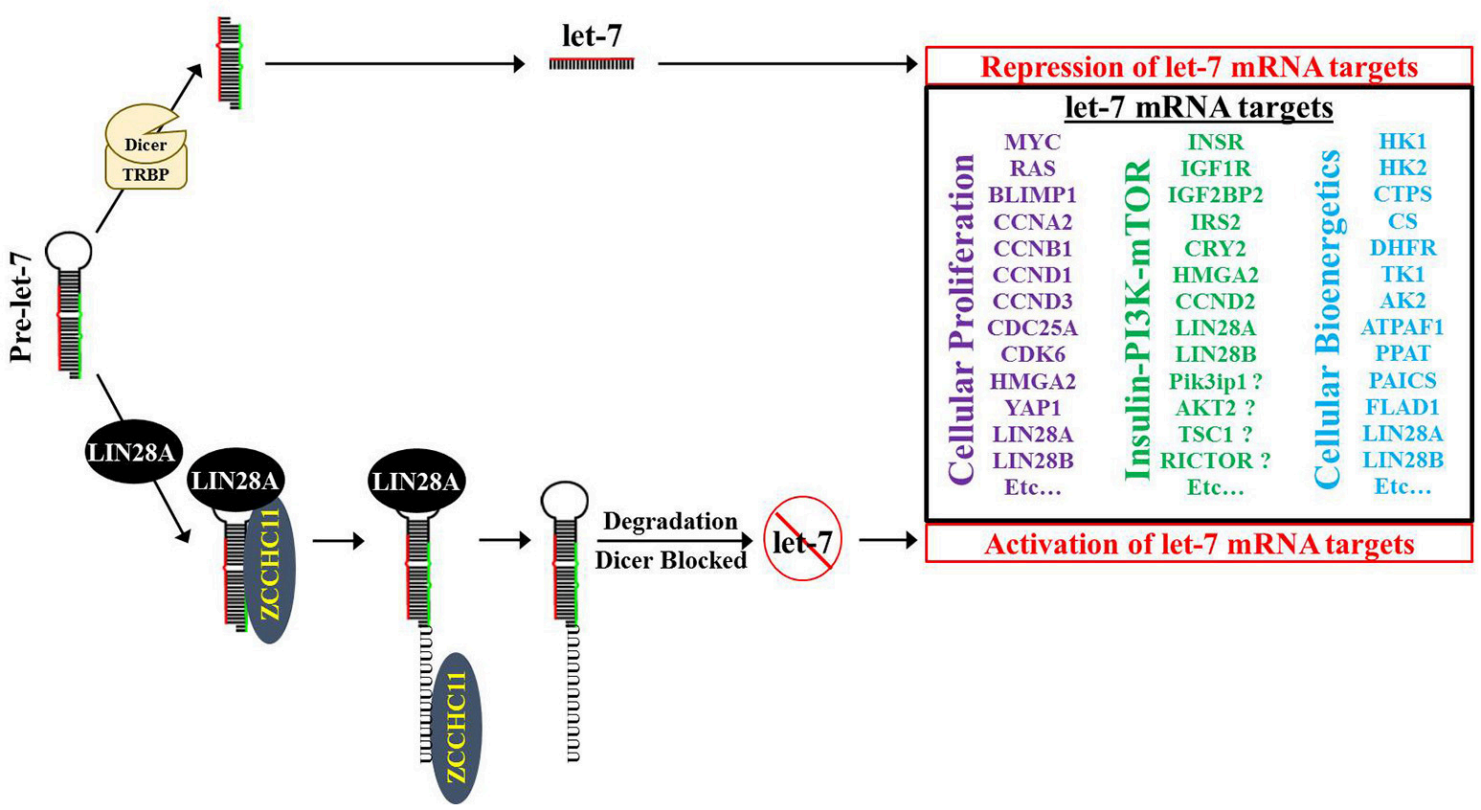
**Model for LIN28A/ZCCHC11 regulation of let-7 microRNA biogenesis, promoting cellular proliferation and modifying bioenergetics**. LIN28A recruits the TUTase ZCCHC11 to pre-let-7 where ZCCHC11 adds a short polyU tail to pre-let-7. Pre-let-7 is no longer a DICER substrate and is targeted for degradation by DIS3L2, thereby blocking let-7 maturation into its functional tumor suppressor form. Loss of mature let-7 microRNAs causes overexpression of numerous oncogenes and bioenergetic genes.

LIN28A and/or LIN28B are implicated in key biological functions such as development, glucose metabolism, and pluripotency via let-7 dependent and independent mechanisms (Zhu et al., 2011; Mayr and Heinemann, 2013; Zhang et al., 2016). Studies have shown that LIN28A is a stem cell pluripotency factor. Using OCT4, SOX2, NANOG, and LIN28A, adult human fibroblasts were successfully reprogrammed into induced pluripotent stem cells (Yu J. et al., 2007). Of note, three of the four Yamanaka factors transactivate LIN28A expression and LIN28A on its own is sufficient for reprogramming (Hanna et al., 2009). Repression of let-7 miRNAs is important in establishing the pluripotent state. Let-7 miRNAs directly repress a pantheon of well-known oncogenes such as RAS, MYC, HMGA2, BLIMP1, among others (Figures 4, 5; Zhou et al., 2013). In addition, LIN28AB is thought to alter cellular bioenergetics and glucose metabolism that has direct cancer implications. The role of LIN28AB in metabolism will be discussed in greater detail later in this review.

## LIN28A and LIN28B block biogenesis of the tumor suppressor let-7 microRNA family

Given the observation that global let-7 microRNA biogenesis was blocked in multiple cellular contexts, several research teams interrogated the responsible mechanism (Heo et al., 2008, 2009; Newman et al., 2008; Rybak et al., 2008; Viswanathan et al., 2008; Hagan et al., 2009; Piskounova et al., 2011). When taken together, the consensus is that cytoplasmic Lin28A first binds to the conserved terminal loop of pre-let-7 and recruits the TUTase Zcchc11 (also known as TUT4) to polyuridylate pre-let-7, thereby blocking Dicer cleavage (Figure 5; Hagan et al., 2009; Heo et al., 2009; Thornton et al., 2012). Polyuridylated pre-let-7 is degraded by the exonuclease Dis3L2, a gene implicated in Perlman Syndrome gene (Chang et al., 2013; Ustianenko et al., 2013; Faehnle et al., 2014). Of note, loss of Dis3L2 does not elevate mature let-7 levels, since polyuridylated pre-let-7 is a very poor Dicer substrate. A recent study suggested that the TUTase Zcchc6 (also known as TUT7) may be partially redundant with Zcchc11 in Lin28A-mediated let-7 repression (Thornton et al., 2012). Specifically, knockdown of Zcchc6 did not change mature let-7 levels on its own; however, knockdown of both Zcchc11 and Zcchc6 led to an increase in let-7 levels in comparison to single Zcchc11 knockdown. In contrast to this result, loss of ZCCHC11 via siRNA on its own was sufficient to cause regression of established LIN28A-positive tumor xenografts to a similar level as LIN28A knockdown, raising doubts on TUTase redundancy in let-7 repression in LIN28A-positive human cancer cells (Piskounova et al., 2011). To date, no other reports have been published that have independently tested TUTase redundancy in LIN28A-mediated let-7 repression.

With regards to suppression of let-7, there are four distinct modes of action that have been hypothesized where multiple mechanisms may be responsible or have differential significance based on cell type. The first model suggested that LIN28B binds pri-let-7 in the nucleus, blocking Microprocessor cleavage (Newman et al., 2008; Viswanathan et al., 2008). A second model is that LIN28B likely binds pre-let-7 in the cytoplasm, blocking Dicer cleavage (Rybak et al., 2008). The third model is that LIN28B directs polyuridylation of pre-let-7 via an unidentified TUTase, blocking Dicer cleavage and leading to degradation of polyuridylated pre-let-7 (Heo et al., 2008). Of note, CLiP-Seq of DIS3L2-associated RNAs identified polyuridylated pre-let-7 in the LIN28B-positive HEK-293T cell line (Ustianenko et al., 2016). The final model demonstrated that LIN28B does not require the TUTase ZCCHC11 to block let-7 biogenesis and proposed that LIN28B blocks let-7 biogenesis by sequestering pri-let-7 in the nucleolus (Piskounova et al., 2011); however, the functional consequence of another TUTase ZCCHC6 was not investigated in this study. This omission is notable as subsequent work from the Gregory lab showed that LIN28B promotes pre-let-7 *in vitro* uridylation by ZCCHC6 (Thornton et al., 2012). Since the subcellular localization of LIN28B is controversial, the precise mechanisms of LIN28B-mediated let-7 repression and whether or not LIN28B utilizes a TUTase for let-7 repression remains unclear.

## Let-7 independent functions of LIN28AB in post-transcriptional gene regulation

Besides let-7 miRNAs, LIN28AB also functions in post-transcriptional gene regulation by directly binding to specific mRNAs leading to altered translational efficiency. For example, Insulin-like growth factor 2 (Igf2) was one of the first mRNAs discovered to be bound by Lin28A (Polesskaya et al., 2007). In skeletal myoblasts, Lin28A enhances Igf2 expression by recruiting Igf2 mRNA to polysomes through interactions with translation initiation complexes. In human embryonic stem cells, LIN28A acts as a post-transcriptional regulator for OCT4, a reprogramming factor. This study demonstrated that LIN28A promoted translation of OCT4 by directly binding within coding region of its mRNA via interaction with RNA helicase A (RHA; Qiu et al., 2010). Other studies using mouse ESCs uncovered the association of Lin28A with more mRNA targets including histone H2a, Cyclin A, Cyclin B and CDK4. Lin28A facilitates the expression of these mRNAs by directly binding to either their coding region (Xu and Huang, 2009) or 3′ untranslated region (3′-UTR; Xu et al., 2009). Interestingly, a recent study conducted by Daley's group found that Lin28A expression enhanced adult tissue repair in a mouse model by reprogramming cellular bioenergetics (Shyh-Chang et al., 2013b). This study showed that let-7 suppression was necessary but not sufficient to improve tissue repair. Enhanced translation of a several metabolic enzymes due to Lin28a was also required. To investigate if there is a consensus LIN28A binding motif in mRNAs and the global impact of LIN28A on mRNA expression, Wilbert and colleagues conducted a study employing crosslinking and immunoprecipitation coupled with high-throughput sequencing (CLiP-Seq) in human ES and somatic cells (Wilbert et al., 2012). It was found that, similar to its interaction with precursor let-7 miRNA transcripts, LIN28A binds to a consensus GGAGA motif enriched within coding exons and 3′-UTR of mRNAs. In the study, a positive feed-forward autoregulation of LIN28A was revealed where LIN28A directly amplifies its own translation by binding to sites within 3′-UTR of its mRNA. Furthermore, LIN28A-mediated post-transcriptional regulation was also responsible for the expression of several RBPs. Increased levels of LIN28A induced widespread changes in alternative splicing indicating LIN28A-mediated regulation of splicing factors. In a recent study, similar results were obtained for LIN28B using photoactivatable-ribonucleoside-enhanced crosslinking and immunoprecipitation (PAR-CLIP) on HEK293 cells (Hafner et al., 2013).

## Clinical relevance and role of LIN28A/LIN28B in cancer

Reactivation of either LIN28A or LIN28B is a hallmark of many human cancers where the expression of these proto-oncogenes is typically mutually exclusive. Table 1 lists multiple studies that have evaluated the diagnostic and prognostic value of LIN28A, LIN28B, and/or let-7 expression in cancer. Altogether, these studies demonstrate the LIN28AB expression and let-7 loss almost invariably correlates with poor prognosis. As tumor suppressors, let-7 miRNAs repress several oncogenes including K-RAS, C-MYC, HMGA2 and cell cycle factors (Cyclin D1, D2; Roush and Slack, 2008). Under pathological conditions, loss of let-7 expression triggers derepression of these oncogenes leading to tumorigenesis or facilitating tumor growth and/or metastasis. LIN28A and LIN28B inhibit biogenesis of all let-7 miRNAs with the possible exception of let-7a-3 (Triboulet et al., 2015), implicating the coordinate downregulation of this tumor suppressor microRNA family in disease. In fact, high levels of LIN28A or 28B are found in many human cancers such as glioblastoma, ovarian, gastric, prostate, and breast cancer (Dahiya and Morin, 2010; Cai et al., 2013; Qin et al., 2014). In cases where a specific cancer type expresses both LIN28A and LIN28B, these tend to occur in distinct tumor subtypes. As an example, HER2+ breast cancer typically expresses LIN28A, while triple negative breast cancer is LIN28B (Piskounova et al., 2011; Yang J. et al., 2015; Shen et al., 2016). A study conducted by Viswanathan and colleagues using primary human tumors and human cancer cell lines showed that about 15% of human cancers displayed high LIN28A/LIN28B and low let-7 expression pattern. Overexpression of LIN28A/LIN28B is related with advanced disease stage among multiple tumor types and typically associates with poor prognosis (Viswanathan et al., 2009). Results from another study showed that LIN28B promotes oncogenesis and tumor progression in head and neck cancer cells by repressing let-7 miRNAs (Alajez et al., 2012). Subsequent activation of let-7 targets such as HMGA2, CCND2, IGF1R, and IGF2BP2 was also validated.

**Table 1:** **Clinical relevance of LIN28A/LIN28B expression in human cancers**.

**Cancer type**	**Sample size**	**Detection method**	**Clinical relevance**	**References**
20 cancer types	Meta-analysis of 46 studies		Of all tumor suppressor microRNAs, let-7 loss most frequently correlated with poor prognosis across cancers	Nair et al., 2012
Acute myeloid leukemia (AML)	108	IHC	LIN28B↑ poorer prognosis	Zhou et al., 2016
Atypical teratoid/rhabdoid tumor	24	IHC, RT-qPCR	19/24 tumors express LIN28A, many tumors also express LIN28B by RT-qPCR	Weingart et al., 2015
Brain cancers (Pediatric)	450	FISH, IHC	LIN28A/B serves as a diagnostic marker for pediatric brain tumors	Spence et al., 2014
Breast cancer	15	IHC	LIN28A↑ higher grade	Viswanathan et al., 2009
Breast cancer	33	IHC, RT-qPCR	9/33 LIN28A-positive (correlates with HER2+), 10/33 LIN28B-positive (correlates with triple negative)	Piskounova et al., 2011
Breast cancer	569	IHC	174/569 had high LIN28A and were characterized by worse prognosis, LIN28A expression associated with HER2+ tumors	Feng et al., 2012
Cervical cancer	11	IHC	LIN28A↑ higher grade	Viswanathan et al., 2009
Chronic myeloid leukemia (CML)	35	RT-qPCR	LIN28A↑ in accelerated phase & blast crisis CML in comparison to chronic phase	Viswanathan et al., 2009
Colon cancer	45	IHC	19/45 LIN28A-positive, 14/45 LIN28B-positive	Piskounova et al., 2011
Colon cancer	228	Tissue microarray, IHC	LIN28B↑ reduced patient survival and higher probability of tumor recurrence	King et al., 2011
Colon cancer	357	IHC	LIN28B↑ reduced survival and higher disease recurrence	Pang et al., 2014
Colorectal cancers	595	IHC	10% LIN28A+, 8% LIN28B, 20% LIN28A+/LIN28B+, LIN28A↑ reduced survival	Tu et al., 2015
Esophageal cancer	161	IHC	Either LIN28A or LIN28B↑ more aggressive tumors and poor prognosis	Hamano et al., 2012
Gastric cancer	239	RT-PCR, IHC	LIN28A↑ poor prognosis	Xu et al., 2013
Gastric cancer	47	IHC	LIN28A as a predictive biomarker for neoadjuvant chemotherapy	Teng et al., 2013
Gastric cancer	298	IHC, RT-qPCR	LIN28A↑ poor prognosis. It can be used as a prognostic factor in chemotherapy	Wang et al., 2014
Gastric cancer	97	IHC	LIN28A↑ poor prognosis	Hu et al., 2014
Germ cell tumors (Extragonadal)	131	IHC	LIN28A as a sensitive marker for primary extragonadal seminoma/germinomas, embryonal carcinoma, and yolk sac tumors with high specificity	Cao et al., 2011b
Glioblastoma multiforme (GBM)	107	Gene microarray, Tissue microarray	LIN28A↑ shortened overall and progression-free survival	Qin et al., 2014
Glioma (pediatric and ^*^adult)	139+ TCGA	IHC, RT-qPCR	LIN28A↑ more common in high grade/GBM, 42% of GBM express either LIN28A or LIN28B	Mao et al., 2013
Liver cancer	89	Microarray	LIN28B↑ higher disease recurrence and associated with advanced stage	Viswanathan et al., 2009
Liver cancer	129	RT-qPCR	LIN28A↑ prognostic factor for poor overall survival	Qiu et al., 2012
Medulloblastoma	90	Microarray	LIN28B↑ and let-7↓ correlated with poor prognosis in Group 3 and Group 4 subtypes	Northcott et al., 2009
Medulloblastoma	238	RNA-Seq	LIN28B↑ and let-7↓ correlated with poor prognosis in Group 3 and Group 4 subtypes	Hovestadt et al., 2014
Multiple Myeloma	542	Microarray	LIN28B↑ poor prognosis	Manier et al., 2016
Neuroblastoma	2817	SNP array, RT-qPCR, microarray	LIN28B↑ poor prognosis	Diskin et al., 2012
Oral squamous cell carcinoma	12	RT-qPCR	Higher expression of LIN28A in the neoplastic samples	Sterenczak et al., 2014
			Increased levels of LIN28B could be associated with poor prognosis OSCCs	
Ovarian primitive germ cell tumors	121	IHC	LIN28A is sensitive diagnostic marker for ovarian germ cell tumors	Xue et al., 2011
Ovarian cancer (Epithelial)	140	RT-qPCR, IHC	High LIN28B and IMP3 is associated with poorer prognosis	Hsu et al., 2015
Ovarian cancer (Epithelial)	211	SNP assay, RT-qPCR	LIN28B↑ higher mortality rate and increased relapse risk	Lu et al., 2012
Ovarian cancer	Multiple datasets	Microarray, RT-qPCR	LIN28B↑ in C5 Ovarian Cancer	Helland et al., 2011
Pheochromo-cytomas and Paragangliomas	208	Tissue microarray	LIN28A expression is associated with mutations in one of the succinate dehydrogenase genes	Oudijk et al., 2015
Primitive neuroectodermal tumor	51	Microarray, IHC, RT-qPCR	LIN28A is a promising prognostic marker	Picard et al., 2012
Primitive neuroectodermal tumors (Supratentorial)	47	IHC	High LIN28A and OLIG2 is associated with poorer prognosis	Choi et al., 2016
Prostate cancer	42	IHC	LIN28B promotes growth and activates androgen receptor	Tummala et al., 2013
Prostate cancer	41	IHC	LIN28AB higher in metastatic tumor than primary, expression inversely correlated with ESE3/EHF	Albino et al., 2016
Testicular germ cell tumor	184	IHC	LIN28A was a highly sensitive diagnostic marker for testicular germ cell tumors	Cao et al., 2011a
Wilms tumor	105	IHC	LIN28B overexpression is related to higher risks of relapse and poor survival	Urbach et al., 2014

**Figure 6:**
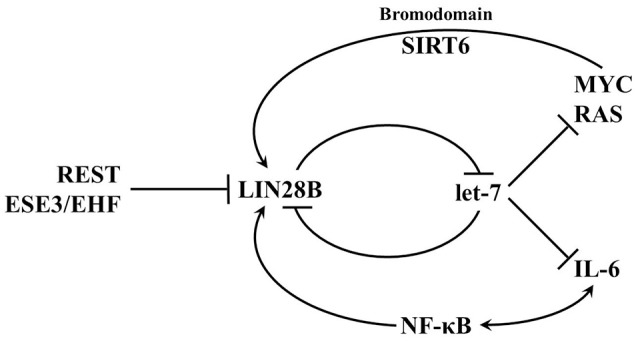
**Transcriptional networks that regulate LIN28B expression**. LIN28B is highly expressed during embryogenesis and as differentiation progresses, LIN28B expression is lost. In adult mammals, only a small subset of somatic cells exist where LIN28B expression occurs. Several transcription factors such as MYC and NF-κB promote LIN28B transcription, while REST and ESE3/EHF are transcriptional repressors.

Activation of LIN28AB expression in cancer cells can be triggered by upstream transcriptional factors and/or loss of transcriptional repressors (Figure 6); however, much remains to be discovered about how transcription of these two genes is regulated. In breast cancer cells, depletion of C-MYC by siRNA decreased LIN28A transcript and protein level, whereas overexpression of C-MYC restored LIN28A expression (Dangi-Garimella et al., 2009). Activation of LIN28B by C-MYC has also been confirmed in multiple human and mouse tumor models (Chang et al., 2009; Jiang et al., 2012). Direct association of c-MYC with LIN28B promoter led to transcriptional transactivation of the protein and LIN28B expression was necessary and sufficient for MYC-mediated Let-7 suppression. These results establish a positive feedback loop between LIN28A/LIN28B and C-MYC. Whereas, LIN28A/LIN28B depresses C-MYC by repressing let-7 miRNAs, C-MYC post-transcriptionally activates expression of LIN28A and LIN28B. In another study, LIN28B was found to link molecularly inflammation and cancer via a positive feedback loop involving NF-κB activation and derepression of the let-7 regulated gene IL-6 (Iliopoulos et al., 2009). In addition, both genetic and epigenetic events contribute to LIN28B expression in a subset of cancers (Viswanathan et al., 2009). In rare cases, LIN28B expression may be activated in part through genomic amplification or in Wilms tumor by a chromosomal translocation. Epigenetic regulation via LIN28B promoter hypomethylation may poise the LIN28B locus for transcriptional activation. In contrast to LIN28AB reactivation in adult cancer, some childhood cancers may be derived from cells that failed to silence LIN28AB expression that acquired additional genetic mutations. These childhood cancers include atypical teratoid/rhabdoid tumors (AT/RT), pediatric CNS-PNET, Wilms tumor, medulloblastoma, and neuroblastoma.

In addition to the LIN28AB/let-7 axis, LIN28AB might also induce oncogenesis through other signaling pathways. For example, a MLL-fusion/MYC/LIN28A–|miR-150–| FLT3/MYB/HOXA9/MEISI signaling pathway was recently identified where LIN28A-mediated inhibition of miR-150 maturation is the pivotal step of leukemogenesis (Jiang et al., 2012). A recent study showed that LIN28A binds to bone morphogenetic proteins 4 (BMP4) mRNA promoting its expression at a post-transcriptional level in ovarian cancer cells (Ma et al., 2013). Overexpression of BMPs increases ovarian CSCs proliferation and promotes tumor growth (McLean et al., 2011). When combined with tumor cells *in vivo*, ovarian carcinoma-associated mesenchymal stem cells enhanced proliferation of ovarian CSCs and tumorigenesis through increased BMPs expression. Furthermore, *in vitro* treatment with exogeneous BMP2 increased growth of ovarian CSCs, whereas *in vitro* and *in vivo* inhibition of BMP signaling partly abrogated mesenchymal stem cell-induced tumor growth (McLean et al., 2011). Thus, the finding provides a new regulation mechanism of ovarian tumor growth by LIN28A and the potential for the development of novel treatment strategies against ovarian cancer. LIN28A was also found to activate the translation of human epidermal growth factor receptor 2 (HER2) and HMGA1 by directly interacting with their mRNA in breast cancer cells (Feng et al., 2012).

## LIN28A/LIN28B in CSCs, metastasis, and resistance to chemotherapy and radiotherapy

Cancer stem cells (CSCs) play a pivotal function in numerous pathological events during tumorigenesis and tumor progression (O'Connor et al., 2014; Takahashi et al., 2014). Many frontline therapies fail to eradicate cancer fully due to their failure to kill CSC and the ability of CSCs to initiate tumor relapse, to promote metastasis, and to confer resistance to chemotherapy and radiation therapy. Therefore, the development of novel therapeutics requires the elucidation of the contributing genetic factors that lead to CSC-dependent aggressive pathology. There is accumulating evidence showing that LIN28A/LIN28B is important in the formation of CSCs in certain cancer types and many consider them as biomarkers for CSCs (Table 2; Viswanathan and Daley, 2010). In addition, LIN28AB expression and let-7 loss has been associated with resistance to numerous cancer therapies (Table 3).

**Table 2:** **LIN28 in cancer stem cells**.

**Cancer type**	**CSC population**	**LIN28A or LIN28B**	**References**
Breast cancer	ALDH1+, high Vimentin, low E-cadherin	LIN28A	Cai et al., 2013
Breast cancer	SOX2+, Nestin+, GFAP+, Synaptophysin	LIN28A	Mao et al., 2013
Breast cancer	ALDH1+	LIN28A	Yang et al., 2010
Breast cancer	IMP2	LIN28A/B	Degrauwe et al., 2016
Breast cancer	High CD24, Low CD44	LIN28B	Iliopoulos et al., 2009
Breast cancer	CD44 +, CD24-	LIN28A	Cai et al., 2013
Colon cancer	LGR5+, PROM1+	LIN28B	King et al., 2011
Lung cancer (Non-small cell)	CD166+	LIN28B	Zhang et al., 2012
Oral carcinoma	IGFBP5+. POSTN+	LIN28A	Hayashi et al., 2013
Ovarian cancer	Oct4+	LIN28A	Peng et al., 2010
Ovarian cancer	ALDH1+	LIN28A	Yang et al., 2010
Ovarian cancer	CD44+, CD24+, Epcam+, Ecadherin-	LIN28A	Meirelles et al., 2012
Pancreatic cancer	ABCG2+, Nestin+	LIN28A	Hamada et al., 2012
Prostate cancer	Sox2+, Nanog+, Oct4+	LIN28B	Kong et al., 2010
Prostate cancer	ESE3/EHF	LIN28A/B	Albino et al., 2016

**Table 3:** **LIN28 and let-7 in resistance to anticancer therapies**.

**Cancer type**	**LIN28 or let-7**	**Treatments**	**References**
Breast cancer	LIN28A	Paclitaxel	Lv et al., 2012
Breast cancer	LIN28A	Radiation	Wang et al., 2013
Breast cancer	let-7a, b, c, d, e, f, g, i	Tamoxifen	Zhao et al., 2011
Breast cancer	let-7b	5-Fluorouracil, Doxorubicin	Wang et al., 2015
Breast cancer	let-7e	Doxorubicin	Lv et al., 2014
Gastric cancer	LIN28A	Oxaliplatin, Paclitaxel, Doxorubicin, Fluorouracil	Teng et al., 2015
Gastric cancer	let-7b	Cisplatin, Vincristine	Yang X. et al., 2015
Glioblastoma multiforme	let-7a, b, c, d, e, f, g, i	Radiation	Chaudhry et al., 2010
Hepatocellular Cancer	LIN28A	Paclitaxel	Tian et al., 2014
Hepatocellular Cancer	let-7g	5-Fluorouracil	Tang et al., 2014
Lung cancer	LIN28A	Radiation	Oh et al., 2010
Lung cancer	LIN28B	Radiation	Jeong et al., 2009
Oral cancer	let-7d	Cisplatin/paclitaxel	Chang et al., 2011
Ovarian cancer	LIN28A	Doxorubicin/Cisplatin	Meirelles et al., 2012
Ovarian cancer	LIN28B	Paclitaxel	Yan et al., 2014
Ovarian cancer	let-7g	Doxorubicin	Boyerinas et al., 2012
Pancreatic cancer	LIN28A	Radiation	Oh et al., 2010
Pancreatic cancer	let-7b, c, d, e	Gemcitabine	Li et al., 2009
Pancreatic cancer	let-7a	Gemcitabine	Bhutia et al., 2013
Prostate cancer	LIN28A	Enzalutamide, Abiraterone, Bicalutamide	Tummala et al., 2016
Renal cell carcinoma	let-7b, c	5-Fluorouracil	Peng et al., 2015

Aldehyde dehydrogenase 1 (ALDH1) is a widely accepted biomarker for CSCs in multiple cancers. A recent study showed that LIN28A expression was positively correlated with a higher percentage of ALDH1 positive cancer cells and is important in maintaining these cells (Yang et al., 2010). In the study, a double-negative feedback loop between LIN28A and let-7 mechanism. Another study provided some intriguing results on the role of LIN28A in the epithelial-to-mesenchymal transition (EMT), an important characteristic of CSCs, and stemness in breast cancer cells (Liu et al., 2013). It was reported that LIN28A significantly induced EMT via down-regulation of let-7 microRNAs including let-7a. Furthermore, LIN28A expression was shown to promote adherence and migration in cancer cells and is correlated with tumor invasiveness. Expression of stem cell factors such as NANOG, SOX2, OCT4, and LIN28B was found to be upregulated in prostate cancer cells with an EMT phenotype (Kong et al., 2010). Down-regulation of LIN28B reduced self-renewal ability and increased let-7 level in these prostate cancer cells. In non-small cell lung cancer, overexpression of LIN28B and glycine decarboxylase, a metabolic enzyme, was required for tumorigenesis and growth of CSCs (Zhang et al., 2012).

NIH-3T3 cells overexpressing either murine Lin28a or human LIN28B generated tumors capable of local invasion in nude mice (Viswanathan and Daley, 2010). There is a growing body of evidence demonstrating that overexpression of LIN28 and/or down-regulation of let-7 contributes to metastasis and tumor resistance to chemotherapy and radiation therapy (Table 3). For example, Raf kinase inhibitory protein (RKIP) negatively regulates the MAP kinase, G protein-coupled receptor kinase-2 and NF-κB signaling cascades. Overexpression of RKIP enhances chemotherapeutic drug-induced apoptosis in cancer cells (Odabaei et al., 2004). In an orthotopic murine breast cancer model, RKIP effectively suppresses invasion by metastatic breast cancer cells *in vitro* and inhibits tumor cell intravasation and bone metastasis *in vivo* (Dangi-Garimella et al., 2009). The underlying mechanism of RKIP-mediated suppression of metastasis involves a MAPK/Myc/Lin28/Let-7/HMGA2 signaling pathway. RKIP was found to repress MAPK expression leading to attenuated transcription of Lin28 by Myc. Inhibition of Lin28 promotes Let-7 expression leading to inhibition of HMGA2 which is a driver of tumor metastasis.

For breast cancer, first-line chemotherapy including treatment with taxanes such as paclitaxel typically induces 30–70% therapeutic response rates. However, many patients develop metastasis and/or tumor relapse after 6–10 months (Seo et al., 2007). Lin28 was found to mediate resistance of breast cancer cells to paclitaxel by modulating p21, Rb, and Let-7a miRNAs (Lv et al., 2012). As compared to cells expressing a low level of LIN28A, T47D cancer cells with high expression of Lin28 was shown to be more resistant to paclitaxel. While stable expression of Lin28 in cancer cells with low Lin 28 levels significantly reduced their sensitivity to paclitaxel, LIN28A knockdown in T47D cells effectively sensitized them to the drug. In another study, LIN28A was reported to be responsible for breast cancer cell resistance to radiation (Wang et al., 2013). Highest expression level of LIN28A was found in T47D cancer cells which were most resistant to radiation treatment. Stable expression of LIN28A not only rendered cancer cells more resistant to radiation, but dramatically inhibited radiation-induced apoptosis. On the other hand, down-regulation of LIN28A expression or overexpression of let-7a miRNA effectively augmented cancer cell sensitivity to radiation. It was proposed by the authors that Lin28 might regulate radioresistance of breast cancer cells through H2A.X and caspase-dependent signaling pathways. In another study, LIN28A/let-7 axis was found to be involved in the resistance of human lung and pancreatic cancer cells with a K-RAS mutation (Oh et al., 2010). Overexpressing let-7a or decreasing LIN28A expression, cancer cells became more sensitive to radiation. Taken together, novel therapies designed to target LIN28AB might help achieve better therapeutic outcomes for the treatment of drug and radiation-resistant tumors and attenuate metastatic lesions.

## LIN28AB and metabolomics

Several recent reports have discovered roles for the LIN28AB/let-7 pathway in metabolism. From 2010, the Lin28AB/let-7 axis was directly implicated in regulation of glucose metabolism in mammals using transgenic overexpression and conditional knockout mice. A mouse strain with doxycycline-inducible Lin28A is characterized by low level, leaky expression in muscle, skin, and connective tissues in the absence of the inducer (Zhu et al., 2010). These mice have increased body size, delayed onset of puberty, insulin insensitivity, and increased glucose metabolism that results from a shift toward glycolysis and elevated lactate production. Increased glucose uptake is attributed to Lin28ab-mediated repression of let-7 and consequent suppression of the Insulin-PI3K-mTOR pathway. In mice with inducible let-7 overexpression, similar effects were found as those observed in Lin28a conditional knockout (Zhu et al., 2011). Increased let-7 expression reduced body size and led to hyperglycemia and glucose intolerance. Whole body overexpression of both Lin28a and let-7 resulted in no overt metabolic defects due to the antagonistic actions of these factors.

Changes in cellular bioenergetics play a central function in tissue repair where Lin28a expression promotes recovery preferentially in young mice (Shyh-Chang et al., 2013b). Specifically, mice ectopically expressing Lin28a in several models of tissue injury such as shaved hair, distal digit amputation, and ear clipping showed the induction of the anagen of the hair, the repair of the digits, and the healing of the ear wounds, respectively. For tissue repair, both let-7 dependent and independent activities of Lin28a are required. The role of Lin28a in tissue regeneration is attributed to elevated glycolysis as well as mitochondrial OxPhos activity resulting from the translational enhancement of metabolic enzymes such as phosphofructokinase, pyruvate dehydrogenase, and isocitrate dehydrogenase.

In cancer cells, the embryonic function of LIN28A/LIN28B is subverted to induce a metabolic shift from oxidative phosphorylation to glycolysis. For example, overexpression of either LIN28A or LIN28B in liver cancer cells promotes the Warburg effect as manifested by elevated glucose uptake, lactate production and oxygen consumption rate, which were reversed upon exposure to let-7 mimetics (Ma et al., 2014). LIN28 meditated post-transcriptional regulation of the metabolic enzyme PDH kinase 1 (PDK1) is thought to contribute to these effects. In mouse ESCs, the Lin28a/let-7 axis regulates the Thr-Gly-SAM pathway (Shyh-Chang et al., 2013a). Overexpression of Lin28a resulted in an increase in Thr-Gly-SAM metabolites while overexpression of let-7 resulted in a concomitant decrease of these metabolites. LIN28A in concert with NANOG, OCT4, and SOX2 can reprogram differentiated cells back into an induced pluripotent state (Yu J. et al., 2007). However, under pathological circumstances, aberrantly expressed LIN28A/LIN28B can stimulate the development of highly aggressive, poorly differentiated tumors. Notably, all reprogramming factors used to transform adult fibroblasts back to IPS cells, have been linked to oncogenesis and many poorly differentiated aggressive human tumors had an embryonic stem-cell like gene expression signature (Ben-Porath et al., 2008; Viswanathan and Daley, 2010). The phenomenon suggests that inducing pluripotency shares commonalities with the aberrant epigenetic reprogramming and alterations in cellular bioenergetics that happens during tumorigenesis (Figures 4, 5).

## Genetically engineered mouse models of LIN28A/LIN28B-expressing cancers

Clinical data and cell culture studies implicate LIN28AB in poor prognosis human cancer. A direct role for LIN28AB in mammalian cancer has been independently confirmed in several studies using genetically engineered mouse (GEM) models. To date, several reported studies have shown that enforced Lin28AB expression is sufficient on its own in select cases to cause cancer and more broadly promotes tumor growth and/or metastasis in existing GEM cancer models. Many of the GEM cancer studies have used mice with inducible Lin28 that were generated by the Daley lab. Specifically, flp-mediated recombination was perfomed in KH2 ES cells, resulting in a single-copy integration downstream of the Col1a1 gene that has a TetO minimal CMV promoter—ORF (either mouse Lin28a or human LIN28B)-pA signal. Therefore, Lin28 transgene expression occurs in the presence of the reverse Tet-transcriptional activator in combination with doxycycline induction. These inducible strains have been used in four cancer studies to date with a focus on colon cancer, liver cancer, Wilms tumor, or mast cell malignancies.

For colon cancer, we performed immunohistochemistry on 45 human tumor samples where 19 were LIN28A-positive while 14 expressed LIN28B (Piskounova et al., 2011). Three independent groups have each analyzed more than 200 colon cancer samples where LIN28AB expression has been shown to be associated with poor prognosis (see Table 1). To investigate a direct role for Lin28ab in mammalian intestinal and colon cancer, the Daley lab used mice with doxycycline inducible Lin28A/LIN28B expression that was limited to cells where Villin-Cre had acted (Tu et al., 2015). They showed that induced Lin28A/LIN28B expression was sufficient to cause tumors restricted to the small intestine. No colon tumors were observed. Induced Lin28ab expression in the context of Apc^Min/+^ mice accelerated tumor development and increased tumor incidence. Strikingly, induced Lin28AB expression led to a substantial increase in colon tumors and the resulting tumors became metastatic relative to Apc^Min/+^ mice that lack Lin28ab. These results clearly show that Lin28AB expression in mouse cancer models can significantly improve their overall quality to mimic human disease.

Many human liver cancers are characterized by LIN28B overexpression. Nguyen and colleagues demonstrated that induced, liver-specific expression of LIN28B was sufficient to cause hepatocellular carcinomoma (Nguyen et al., 2014). In addition, they showed that an inducible Albumin-MYC mouse model for liver cancer has 50% mortality by ~40 days post-induction; however, genetic ablations of both Lin28a and Lin28b in the liver resulted in ~70% of Albumin-MYC mice surviving to 150 days post-induction, the time at which the study terminated. Similarly, Lin28b deletion extended survival in another liver cancer mouse model, LAP-MYC. As another example, a significant proportion (~15%) of Wilms tumor, the most common pediatric kidney cancer, is characterized by LIN28B expression (Urbach et al., 2014). These authors further showed that LIN28B expression in human Wilms tumor correlates with higher relapse risk and poor prognosis and that induced LIN28B expression in the embryonic mouse kidney caused this disease. In addition to the described research that was performed by or in collaboration with the Daley lab, other research teams have investigated Lin28b in cancer using transgenic mice. For example, transgenic mice with targeted LIN28B overexpression in the sympathetic adrenergic lineage, hematopoietic tissues, and intestinal epithelium caused neuroblastoma, peripheral T-cell lymphoma (PTCL), and intestinal/colon adenocarcinoma, respectively (Beachy et al., 2012; Molenaar et al., 2012; Madison et al., 2013). In neuroblastoma cells, LIN28B blocks neuronal differentiation and upregulates MYCN post-transcriptionally by blocking the let-7 microRNAs. In the PTCL model, LIN28B led to upregulation of IL-6, a let-7 microRNA target (Beachy et al., 2012), and is consistent with earlier work from the Struhl lab that linked molecular inflammation and cancer through a positive feedback loop involving NF-κB, LIN28B, let-7, and IL-6 (Iliopoulos et al., 2009).

A major deficiency in the current GEM models is that there is none for LIN28AB-positive cancers that primarily affect women. As Tables 1, 3 illustrate, LIN28AB expression occurs frequently in ovarian cancer and seems responsible in part for the development of drug resistant tumors. In addition, LIN28AB is also significant in breast cancer. Specifically in 33 breast cancer tumors that we interrogated by immunohistochemistry, 9 were LIN28A+, 10 were LIN28B+, and none were positive for expression of both genes (Piskounova et al., 2011). Let-7 levels were negatively correlated with LIN28AB expression as expected in these samples. Furthermore, LIN28A and LIN28B expression is correlated with HER2-positive and triple negative breast cancer, respectively. Multiple research groups have independently validated this result using immunohistochemical methods and analysis of TCGA breast cancer samples (Feng et al., 2012; Xie et al., 2014; Shen et al., 2016). To date, no GEM models have been reported to date that faithfully recapitulate LIN28A expression in HER2+ or LIN28B in triple negative breast cancer. These preclinical mouse models are essential to understand how LIN28AB contributes to disease *in vivo* and to test novel, molecularly targeted drugs.

## Biochemical screens for inhibitors of LIN28AB or the LIN28A-interacting TUTase ZCCHC11

Considering the oncogenic role of LIN28AB, it is evident that inhibition of the LIN28AB/let-7 pathway is an attractive molecular target for cancer chemotherapy. Four high throughput screens have been reported whose objective is the identification of pharmacologically active small molecules that disrupt LIN28AB-mediated let-7 repression. Lin and Gregory used a biochemical assay to find small molecule inhibitors of the mouse TUTase Zcchc11 (Lin and Gregory, 2015a). They screened 14,822 compounds and identified 91 hits in the primary screen. Many of the identified compounds were non-specific, thiol-containing inhibitors as their screen was done in the absence of reducing agents such as dithiothreitol or β-mercaptoethanol. Of the candidate inhibitors that survived biochemical scrutiny under reducing conditions, aurothioglucose hydrate was further validated to block pre-let-7 uridylation *in vivo*. In contrast to this screen that looked for a TUTase inhibitor, the other reported screens sought to identify compounds that block the ability of LIN28AB to bind RNA. Roos et al. used a protein/RNA FRET assay to test ~16,000 compounds for molecules that blocked the LIN28B/let-7 interaction (Roos et al., 2016). They identified a compound N-methyl-N[3-(3-methyl[1,2,4]triazolo[4,3-b]pyridazin-6-yl)phenyl]acetamide that phenocopied LIN28B siRNA in the restoration of let-7 levels in the Huh7 liver cancer cell line. Lightfoot et al. developed a biophysical high throughput screening assay to detect small molecule inhibitors of the LIN28A-pre-let-7 interaction (Lightfoot et al., 2016). They screened a library of 2,768 pharmacologically active compounds and found two compounds 6-hydroxy-DL-DOPA, a dopamine precursor, and SB/ZW/0065, a benzo[a]phenoxazine derivative validated *in vitro*. Lim et al. also used a FRET-based high-throughput screen to find molecules that disrupt the interaction between LIN28A and pre-let-7a (Lim et al., 2016). They screened an in-house library of 4,500 drug-like compounds and identified a benzopyaranylpyrazole-based compound (Compound 1) as a lead hit. This compound upregulated the levels of the let-7 family members in the human choriocarcinoma cell line, JAR as well as PA-1 cells. Furthermore, in JAR cells, exposure to Compound 1 resulted in a reduction of the let-7 targets c-Myc, HMGA2 and Ras. Overall, these screens have successfully identified small molecules that upon further optimization and refinement may provide therapeutic benefit to patients bearing LIN28A/B tumors and improve prognosis. Since this research is in its infancy, no clinical trials are underway interrogating LIN28AB and/or TUTase inhibitors.

## The LIN28AB/let-7 pathway as a molecular target for novel anticancer therapeutics

Conventional cancer treatments including surgery, chemotherapy, and radiation therapy have limited effect against metastatic tumors, drug-resistant recurrent tumors, and CSCs. Novel strategies that can better address these problems are urgently required. Overexpression of LIN28A/LIN28B that results in dysregulation of let-7 microRNAs are essential pathological events in tumorigenesis and progression of many human cancers. The fundamental roles of LIN28A/LIN28B in CSCs, metastasis, resistance to chemotherapy and radiation therapy make them emerging molecular targets for novel anticancer treatments. It is conceivable that correcting aberrant LIN28AB/let-7 signaling pathway will help overcome shortcomings of current anticancer treatments. In mammals, LIN28A and LIN28B are not expressed in adult somatic tissues with few exceptions such as expression in some cardiac and skeletal muscle cells (Yang and Moss, 2003). Evidence from Lin28a and Lin28b knockout mice showed that ablation of either gene in 6-week old mice does not cause overt phenotypes such as differential growth (Shinoda et al., 2013). Accordingly, therapeutics such as shRNAs, siRNAs, and chemical compounds that downregulate LIN28A or LIN28B expression or functional activity are unlikely to have harmful side effects in patients. As mentioned earlier, there is already evidence that downregulation of LIN28A in chemotherapy and radiation-resistant breast cancer cells enhanced their sensitivity to the treatments (Lv et al., 2012; Wang et al., 2013). Suppression of LIN28A by shRNA in human glioblastoma cells caused cell cycle arrest in the G1 phase, enhanced apoptosis, and delayed cell proliferation (Qin et al., 2014). Tristetraprolin (TTP) is an AU-rich element binding protein that functions to regulate gene expression by binding to AU-rich elements in the 3′-UTR of mRNA transcripts and promoting degradation (Blackshear, 2002). Dysregulation of TTP expression and activation of proto-oncogenes coexist in various cancers (Brennan et al., 2009). Intriguingly, TTP was shown to increase levels of mature let-7a, let-7b, let-7f, and let-7g miRNA by post-transcriptionally inhibiting LIN28A expression in ovarian cancer cells. Overexpression of TTP in PA1 ovarian cancer cells successfully suppressed cell growth via let-7b-meidated inhibition of CDC34, which is a downstream target gene of the let-7 family (Kim et al., 2012). In another study, depletion of LIN28A by shRNA reduced *in vitro* invasion ability of bone metastatic breast cancer cells (Dangi-Garimella et al., 2009). Furthermore, their *in vivo* study showed that the metastatic ability of LIN28A-depleted breast cancer cells was significantly impaired.

It is also a feasible to increase let-7 level by interfering with binding of LIN28A/LIN28B to pre-let-7. LIN28AB inhibits let-7 maturation by first interacting with the terminal loop of let-7 precursors. Thus, any small molecules such as aptamers which can effectively block the binding of LIN28AB to immature let-7 will increase let-7 levels. Both *in vitro* and *in vivo* studies have shown that maturation of 7S21L, a chimeric pri-let-7g stem and with the miR-21 terminal loop, is not regulated by LIN28AB, highlighting the fact that the loop is the primary RNA determinant for the LIN28AB interaction (Piskounova et al., 2008; Zhu et al., 2011). Coexpression of 7S21L with LIN28AB in NIH/3T3 cells successfully nullified the ability of LIN28AB to transform cells suggesting let-7 loop mutants might be effective in treating cancers caused by increased level of LIN28AB (Viswanathan et al., 2009). In addition, a truncated stem loop short RNA duplex which has a similar structure to let-7 miRNAs might also help increase let-7 level by acting as a competitive binding inhibitor for LIN28AB. Moreover, synthetic let-7 mimic elicited remarkable anticancer effects on mice human lung cancer xenografts. Treatment with a single dose of let-7b mimic immediately before tumor implantation successfully delayed tumor formation. In addition, intratumoral injections of the synthetic miRNA on established mice tumors also blocked tumor growth (Esquela-Kerscher et al., 2008). In another study, *i.v*. injection of synthetic let-7b conjugated with a neutral lipid emulsion successfully reduced lung tumor burden in the K-RAS orthotopic non-small cell lung cancer mouse model (Trang et al., 2011). Since LIN28A-mediated inhibition of let-7 requires ZCCHC11 (Hagan et al., 2009; Heo et al., 2009; Piskounova et al., 2011), small molecules that inhibit functions of this TUTase may also produce therapeutic benefits in cancer patients. For example, knockdown of ZCCHC11 by siRNA or shRNA increased the level of mature let-7 microRNAs in both mouse embryonic stem cells and human LIN28A-positive cancer cells. Of note, loss of either LIN28A or ZCCHC11 led to regression of established human xenograft tumors.

LIN28A/LIN28B plays an important role in the formation of CSCs. Thus, chemical or small molecular compounds specifically targeting LIN28/let-7 axis might be more potent in eradicating CSCs than conventional chemotherapeutics. Metformin, a diabetes drug, was found to significantly increase let-7a level in CSCs, reverse cancer cell stemness, and markedly improve *in vivo* therapeutic effect of chemotherapeutic (McCarty, 2012). It was hypothesized that the drug might induce these anti-CSCs functions through AMP-activated protein kinase (AMPK)-mediated up-regulation of let-7 miRNAs leading to suppression of LIN28A/LIN28B expression. However, more studies are required to investigate the accuracy of the theory.

In fact, some chemotherapeutics, instead of killing CSCs, can actually enrich CSC populations in tumors. For example, treatment with 5-fluorouracil or oxaliplatin at clinically relevant doses on human metastatic colon cancer cells led to 16- to 30-fold enrichment of CD133+ cell population and 2-fold enrichment of CD44+ cell population. In terms of double-positive (CD133+/CD44+) CSCs, 5- to 22-fold population enrichment was observed (Dallas et al., 2009). In a separate study, treatment with doxorubicin on CD44, CD24, Epcam positive, and E-cadherin negative ovarian CSCs enhanced their ability to form colonies and proliferate (Meirelles et al., 2012). Whereas chemotherapy selectively promotes survival of breast tumor-initiating cells (BT-ICs), enforced expression of let-7 in drug-resistant BT-ICs markedly impaired self-renewal ability of the cells. More importantly, treatment with let-7 not only inhibited tumor growth, but markedly reduced metastasis to the lung and liver of tumor-bearing mice (Yu F. et al., 2007). Consistent with this idea, Mirna Therapeutics, Inc. has a method for let-7 restoration using a chemically modified let-7 microRNA duplex in preclinical development. Since let-7 is widely and abundantly expressed in most cells, it would seem less likely to have off-target effects in comparison to miR-34 replacement therapy. Ultimately, specific targeting of microRNA replacement therapy directly to cancer cells would provide the highest chance for success. To conclude, novel therapeutics which can effectively reduce LIN28AB expression/activity or increase mature let-7 levels have significant potential to improve therapeutic outcomes in LIN28AB-expressing cancer in general and specifically, in metastatic and drug-resistant disease.

## Concluding remarks

Lin-28 and the microRNA let-7 were initially discovered in *C. elegans* as heterochronic genes that regulate developmental timing. In mammals, LIN28A rose to prominence as one of four Thomson reprogramming factors used to generate human induced pluripotent stem cells. LIN28A and its paralog LIN28B are highly related RBPs and proto-oncogenes whose expression is largely restricted to development with particularly high levels in embryonic stem cells. LIN28A and LIN28B exert their biological functions through repressing the biogenesis of the tumor suppressor let-7 microRNA family as well as modifying the translation efficiency of mRNAs that it binds. In >15% cancers, either LIN28A or LIN28B is reactivated. As a result, let-7 is downregulated, leading to enhanced expression of notable let-7 targets such as RAS, MYC, and HMGA2 as well as changes in cellular metabolism. For many cancers that appear not to express LIN28AB in the tumor, the CSC population is LIN28A or LIN28B positive. This population is refractory to many therapies and is thought to give rise to recurrent and more deadly disease. In support of this assertion, LIN28AB via a let-7 dependent mechanism promotes metastasis and resistance to several frontline cancer treatments including ionizing radiation and multiple chemotherapies such as taxanes and platinum-based drugs. It is therefore not surprising that LIN28 expression correlates with poor prognosis.

We and others have provided proof-of-principle experiments that LIN28AB are *bona fide* candidates for cancer intervention. As LIN28AB expression is limited predominantly to tumor cells and conditional mouse knockout of either Lin28a or Lin28b at 6 weeks of age yields no overt phenotypes, it is anticipated that therapeutics targeting the LIN28/let-7 pathway will have minimal side effects in patients. We showed that inhibition of LIN28A or its interacting TUTase ZCCHC11 via siRNA treatment caused regression of established xenograft human tumors in mice. This result was phenocopied by application of a let-7 mimic. Further work revealed that LIN28B represses let-7 by a distinct mechanism that does not rely on ZCCHC11. Inhibition of LIN28B also caused the regression of established xenograft human tumors in mice. Future studies are still needed to define the precise mechanism(s) that are responsible for LIN28B-mediated let-7 repression.

Thus, LIN28 is an attractive molecular target for chemotherapy with the potential to provide benefit to patients afflicted with a subset of poor prognosis cancers. High throughput biochemical screens recently demonstrated that small molecules exist that can modulate the LIN28/let-7 pathway. In the future, larger screens with subsequent chemical optimization of hits hold promise for further pre-clinical drug development. Future research endeavors will reveal not only a more precise role of LIN28 in human disease but also bring to the forefront an exciting frontier in cancer therapeutics as the LIN28/let-7 pathway is exploited for the development of a novel class of LIN28 and/or TUTase inhibitors.

## Author contributions

JB, MM, SC, and JPH were responsible for writing and editing this review article. JB, MM, and SC contributed equally to this review and are co-first authors.

## Funding

This paper was supported in part by a Department of Defense Ovarian Cancer Research Program Pilot Award to JPH.

### Conflict of interest statement

The authors declare that the research was conducted in the absence of any commercial or financial relationships that could be construed as a potential conflict of interest.
